# Regional and Gender Study of Neuronal Density in Brain during Aging and in Alzheimer's Disease

**DOI:** 10.3389/fnagi.2016.00213

**Published:** 2016-09-13

**Authors:** Eva Martínez-Pinilla, Cristina Ordóñez, Eva del Valle, Ana Navarro, Jorge Tolivia

**Affiliations:** Departamento de Morfología y Biología Celular, Facultad de Medicina, Instituto de Neurociencias del Principado de Asturias, Universidad de OviedoOviedo, Spain

**Keywords:** age, Alzheimer's disease, sexual dimorphism, human, hippocampus, entorhinal cortex, frontal cortex

## Abstract

**Background:** Learning processes or language development are only some of the cognitive functions that differ qualitatively between men and women. Gender differences in the brain structure seem to be behind these variations. Indeed, this sexual dimorphism at neuroanatomical level is accompanied unequivocally by differences in the way that aging and neurodegenerative diseases affect men and women brains.

**Objective:** The aim of this study is the analysis of neuronal density in four areas of the hippocampus, and entorhinal and frontal cortices to analyze the possible gender influence during normal aging and in Alzheimer's disease (AD).

**Methods:** Human brain tissues of different age and from both sexes, without neurological pathology and with different Braak's stages of AD, were studied. Neuronal density was quantified using the optical dissector.

**Results:** Our results showed the absence of a significant neuronal loss during aging in non-pathological brains in both sexes. However, we have demonstrated specific punctual significant variations in neuronal density related with the age and gender in some regions of these brains. In fact, we observed a higher neuronal density in CA3 and CA4 hippocampal areas of non-pathological brains of young men compared to women. During AD, we observed a negative correlation between Braak's stages and neuronal density in hippocampus, specifically in CA1 for women and CA3 for men, and in frontal cortex for both, men and women.

**Conclusion:** Our data demonstrated a sexual dimorphism in the neuronal vulnerability to degeneration suggesting the need to consider the gender of the individuals in future studies, regarding neuronal loss in aging and AD, in order to avoid problems in interpreting data.

## Introduction

Compelling evidences of changes in the human brain, at the anatomical and molecular level, related with aging and during Alzheimer's disease (AD) have been described by different authors. Anatomically, it has found a clear decrease in the volume and weight of the brain and histologically an increase in the size of astrocytes and microglia, as well in the neuronal lipofuscin content (Mrak et al., 1997; Sheffield and Berman, 1998; Schultz et al., 2004; Dorszewska, 2013). However, there is a great controversy about the occurrence and extent of neuronal loss processes in these situations. Some influential papers from as early as the 1950s, looking for changes in neuronal density in two-dimensional space concluded that a substantial loss of neurons occurs with age. The percentage of this loss varies from 10 to 60% depending on the methodology employed and the neuronal population examined. Moreover, it has been shown that this death is cell type-dependent since some populations do not exhibit signs of degeneration and others, as cerebral cortex and hippocampus, are particularly affected (Brody, 1955; Colon, 1972). In AD, neuronal loss constitutes one of the major pathological markers that extensively affect different brain areas as entorhinal or prefrontal cortex. This decrease in the neuronal population that reaches 90% correlates well with the severity of the disease (Terry, 2006; Zilkova et al., 2006; Padurariu et al., 2012). The development of more accurate procedures for counting neurons over the last years confirmed these previous observations and open new avenues to better understand how the brain changing with age and with the progression of different neurodegenerative diseases.

The application of stereological techniques to several species, including humans, has led to the conclusion that the decline in neuronal number during aging is not significant in brain regions such as neocortex or hippocampus (West and Gundersen, 1990; Pakkenberg and Gundersen, 1997; Hof and Morrison, 2004) or in some vestibular nuclei (Alvarez et al., 2000). However, it has been reported a highly significant correlation between loss of neurons and age in the entorhinal cortex (Simic et al., 2005) and in the human medial vestibular nucleus (Alvarez et al., 1998). In AD patients, the loss of neurons takes place primarily in the neocortex, hippocampus and entorhinal region (Terry, 2006). Interestingly, the characteristic degenerative processes of AD do not affect equally all cell types. As an example, the pyramidal cells in the entorhinal cortex and the CA1 and *subiculum* regions of the hippocampus seem to be more vulnerable to neurofibrillary tangles (NFT) formation and neurodegeneration than cells of other hippocampal areas (Adachi et al., 2003; Kerchner et al., 2010; Padurariu et al., 2012). The same phenomenon has been observed in the neocortex. This specific vulnerability to degeneration assumes particular importance in the pyramidal cells that furnish long cortico-cortical projections, leading to a global disruption of interconnections among association cortices (Desikan et al., 2010). In contrast, primary sensory and motor areas exhibit minimal loss of neurons.

Several authors have described evidences for sexual dimorphism in the number and cell density in the central nervous system (CNS). In fact, studies on cerebral cortex show that men have 15% more cortical neurons and 13% greater total neuronal density than women, without variations in thickness (Pakkenberg and Gundersen, 1997; Rabinowicz et al., 2002). Complementary studies achieved by magnetic resonance, confirmed that some brain areas such as human cerebellar cortex or gray matter of the left amygdala (Raz et al., 1997) exhibit larger volumes in men than in women (Raz et al., 2004; Sowell et al., 2007). On the contrary, women show larger right striatal and bilateral hippocampal gray matter volumes than men (Neufang et al., 2009). Another sexual dimorphic brain structure in humans is the *corpus callosum*, which is higher in women than in men (DeLisi et al., 1989). All these gender differences may underline gender-specific abilities and susceptibilities to disease, probably influenced by gonadal hormones i.e., estrogen, testosterone and progesterone, in certain areas of the brain. The sexual differentiation of a particular brain region is related to its hormonal environment that comprises local and circulating hormones of the CNS (Neufang et al., 2009). Thus, it seems reasonable to expect that the loss of neuronal cells that occurs in the aging processes as well as in several pathologies of the CNS as AD is influenced by these sexual differences.

In recent years, some studies have been interested in the potential impact of sex on age-related brain changes and in the development of different neurodegenerative diseases. As a rule, these studies concluded that men exhibited greater age-related brain atrophy than women over the entire life expectancy (Gur et al., 1999); this effect is enhanced in the frontal and temporal lobes (Raz et al., 1997; Gur et al., 2002). Meanwhile, a significant reduction of gray matter in women has been reported in the parietal lobes and hippocampus (Raz et al., 2004; Sowell et al., 2007). According with different authors, there is a higher prevalence and incidence of AD in women than in men (Breitner et al., 1988; Fratiglioni et al., 1997; Viña and Lloret, 2010; Alzheimer's Association, 2015). It has been postulated that estrogen deficiency, following menopause, may contribute to the etiology of the disease. In a 12 case-control and cohort studies conducted in the 1990s, it was suggested that estrogen therapy could delay the onset or contribute to the prevention and/or significantly attenuation of AD (Sundermann et al., 2006), but this question is still under discussion (Asthana et al., 1999; Henderson et al., 2000; Mulnard et al., 2000; Henderson, 2014).

The bulk of evidence suggests that sexual dimorphism in the neuronal content may determine the way that aging and neurodegenerative processes affect men and women brains. The aim of this research work is to study the changes in neuronal density in hippocampus and entorhinal and frontal cortices of both, men and women, in order to analyze the possible gender influence during normal aging and in AD.

## Materials and methods

### Human tissues

Use of human brain tissues were approved by “Comité Ético de Investigación Clínica Regional del Principado de Asturias” as follows. These studies were granted waivers of consent on the following bases: (1) samples were gathered retrospectively from pathology archives of necropsies performed for diagnostic purposes; (2) patient identities were anonymized and completely delinked from unique identifiers; and (3) there was no risk to the participants.

Human brain tissues were provided by The Pathologic Anatomy Service of the University Central Hospital of Asturias and the Bank of Neurologic Tissues of the Clinic Hospital of Barcelona. This material was the same used in recent studies of our group (Ordóñez et al., 2012; Martínez et al., 2013; Navarro et al., 2013). Seventy-two cases were employed. Thirty-six individuals with not known neurological, psychiatric, or neuropathological disorders (18 men and 18 women) were divided in three groups according to their age: the first group includes individuals in their 30's and 40's; the second from those in their 50's to the ones in their 70's; and the last one those older than 80. Other thirty-six cases of AD (18 men and 18 women) were divided in three groups based on their AD neuropathological stage, according to Braak's criteria (Braak and Braak, 1991). Postmortem intervals ranged between 2 and 6 h. The pieces from human frontal cortex (Brodmann's area 9), hippocampus and adjacent entorhinal cortex were fixed by immersion in 10% buffered formalin. After fixation, they were washed in distilled water, dehydrated through successive alcohols, cleared in two baths of butyl acetate, embedded in paraffin, and placed in a suitable mold. Transverse sections about 10 μm thick were obtained and attached to gelatin-covered slides, deparaffined in xylene, and rehydrated.

### Neurons, senile plaques, neurofibrillar tangles, and amyloid-beta staining

Alternated sections were stained using a Nissl-like method developed in our laboratory to counterstaining paraffin sections stained with alcoholic Congo Red to show amyloid. This method allows a clear discrimination between neurons and the rest of nervous cells (Navarro et al., 1999). To visualize the typical cerebral markers of the AD neuropathology, the silver technique of Reusche (1991) and a modification of the Congo red method developed in our laboratory (Navarro et al., 1999, 2013) were used.

### Neuronal counting

The estimation of the total number of neurons was carried out using the optical fractionator principle. Stereological analysis was performed using an Olympus BX-51 microscope with Olympus CAST system version 2.0 (Olympus, Denmark A/S, Albertslund, Denmark). The analyzed area (field included by a 20x lens), was delimited. From a random start position, a counting frame was superimposed on the image and neurons were systematically sampled using a 60x lens (Plan Apo N 60x /1.42 oil, Olympus) and the nucleolus as the sampling unit. The sampling frequency was chosen by adjusting the xy-axis step length so that up to 200 cells were counted in each specimen.

Neuronal counting was undertaken on five sections at different levels (separated by 100 μm). For each section, the neuronal density was calculated by dividing the total number of neurons by the area of the sections surveyed. The mean value of each brain region studied was used for the statistical analysis. Hippocampal variations in neuronal density was assessed separately in each anatomical region (CA1, CA2, CA3, CA4; Supplementary Figure 1).

### Statistical analysis

The data in the graphs are presented as the mean ± SD. All statistical calculations were conducted using SPSS 15.0 for Windows. The test of Kolmogorov–Smirnov with the correction of Lilliefors was used to evaluate the fit of the data to a normal distribution. One-way ANOVA followed by multiple comparisons Tukey's test was performed to analyze changes in neuronal density between the different groups of age or the Braak's stages of AD. The Student's *t*-test (two-tailed) was used to analyze possible gender differences. Finally, a Pearson Correlation analysis was used to evaluate the relationship between neuronal density and age or Braak's stage. *p* < 0.05 was defined as statistically significant.

## Results

### Changes in neuronal density during aging

**Figure 1:**
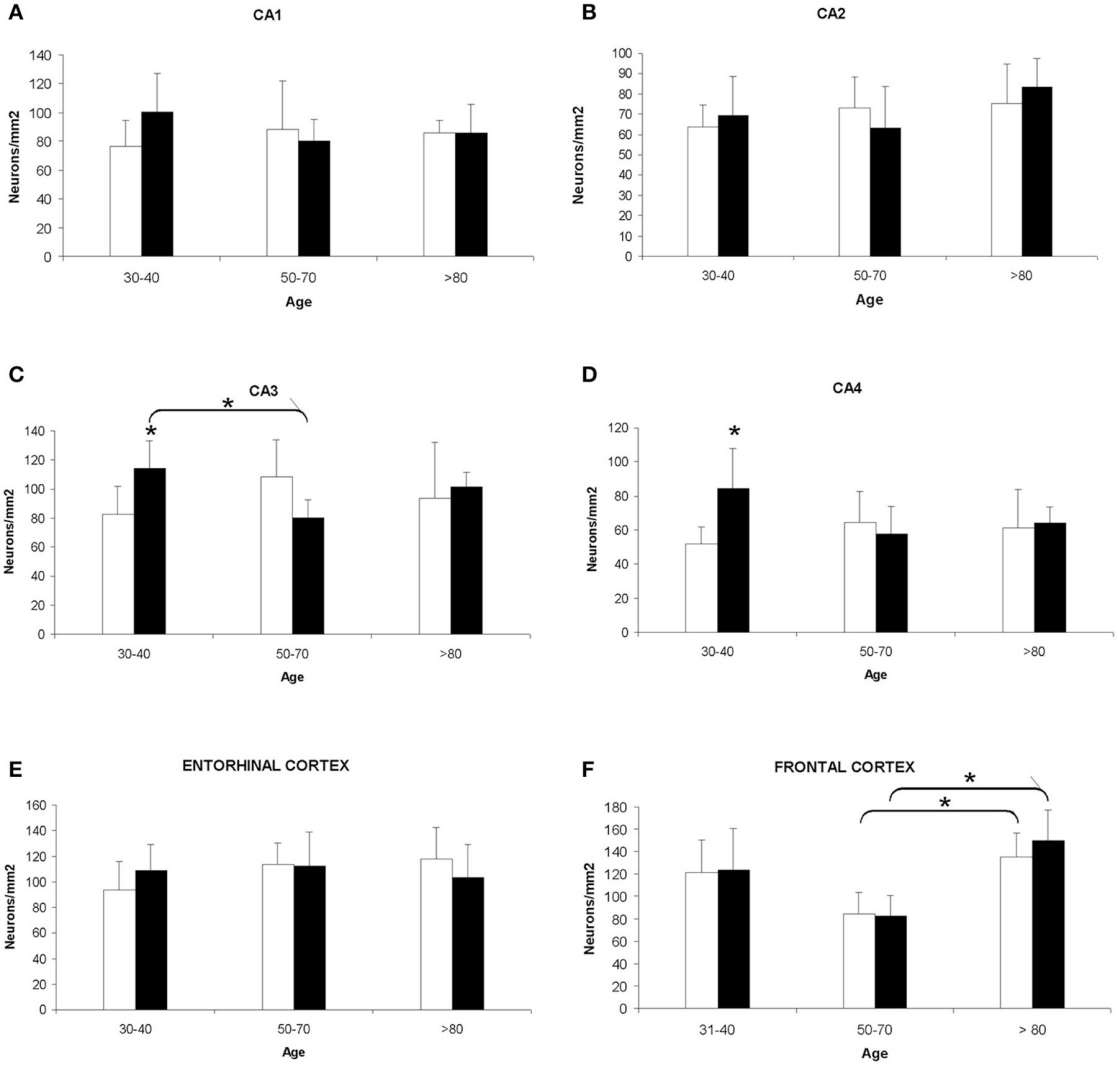
**Quantitative changes in neuronal density in certain hippocampal fields (CA1–4) and in frontal and entorhinal cortices between men and women during aging**. **(A)** CA1 hippocampal area. **(B)** CA2 hippocampal area. **(C)** CA3 hippocampal area. **(D)** CA4 hippocampal area. **(E)** Entorhinal cortex. **(F)** Frontal cortex. Bars represent mean density in a 20x field ± *SD* (*n* = 6). ^*^Statistically significant differences, *p* ≤ 0.05. Women, white bars; Men, black bars.

**Figure 2:**
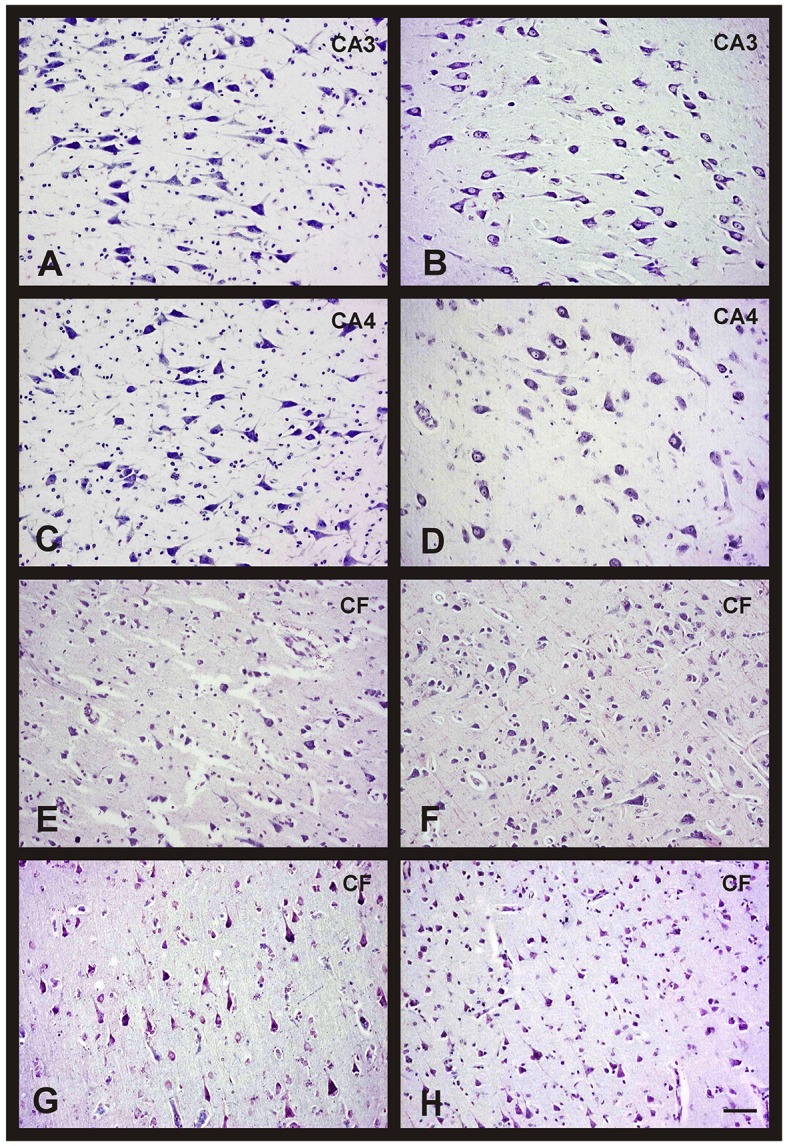
**Changes in neuronal density in hippocampus and frontal cortex between men and women during aging**. Representative microphotographs of human brain sections of non-pathological individuals contrasted with a Nissl method modification. **(A)** Hippocampus (CA3) of a 37 years old man. **(B)** Hippocampus (CA3) of a 35 years old woman. **(C)** Hippocampus (CA4) of a 37 years old man. **(D)** Hippocampus (CA4) of a 35 years old woman. **(E)** Frontal cortex of a 40 years old man. **(F)** Frontal cortex of a 65 years old man. **(G)** Frontal cortex of a 37 years old woman. **(H)** Frontal cortex of a 69 years old woman. Bar, 60 μm.

The quantitative study showed that there were no significant differences regarding neuronal density in CA1, CA2, and entorhinal cortex between women and men, and between groups of age (Figures 1A,B,E). However, the CA3 and CA4 hippocampal areas of the youngest group showed a higher value in men than in women (Figures 1C,D, 2A–D). These differences disappeared with age since the men group tends to suffer a decrease in neuronal density. A correlation between neuronal density and the age of subjects was not found in these areas; only a statistical significant diminution in neuronal density in men of median age was observed respect to the youngest group. In frontal cortex, both sexes showed a decrease in neuronal density in the 50's–70's and a significant increase in elderly with respect to median age subjects (Figures 1F, 2E–H). However, no correlation between the number of neurons per area and age was found in this brain area.

### Morphological changes of hippocampus and entorhinal cortex during aging

**Figure 3:**
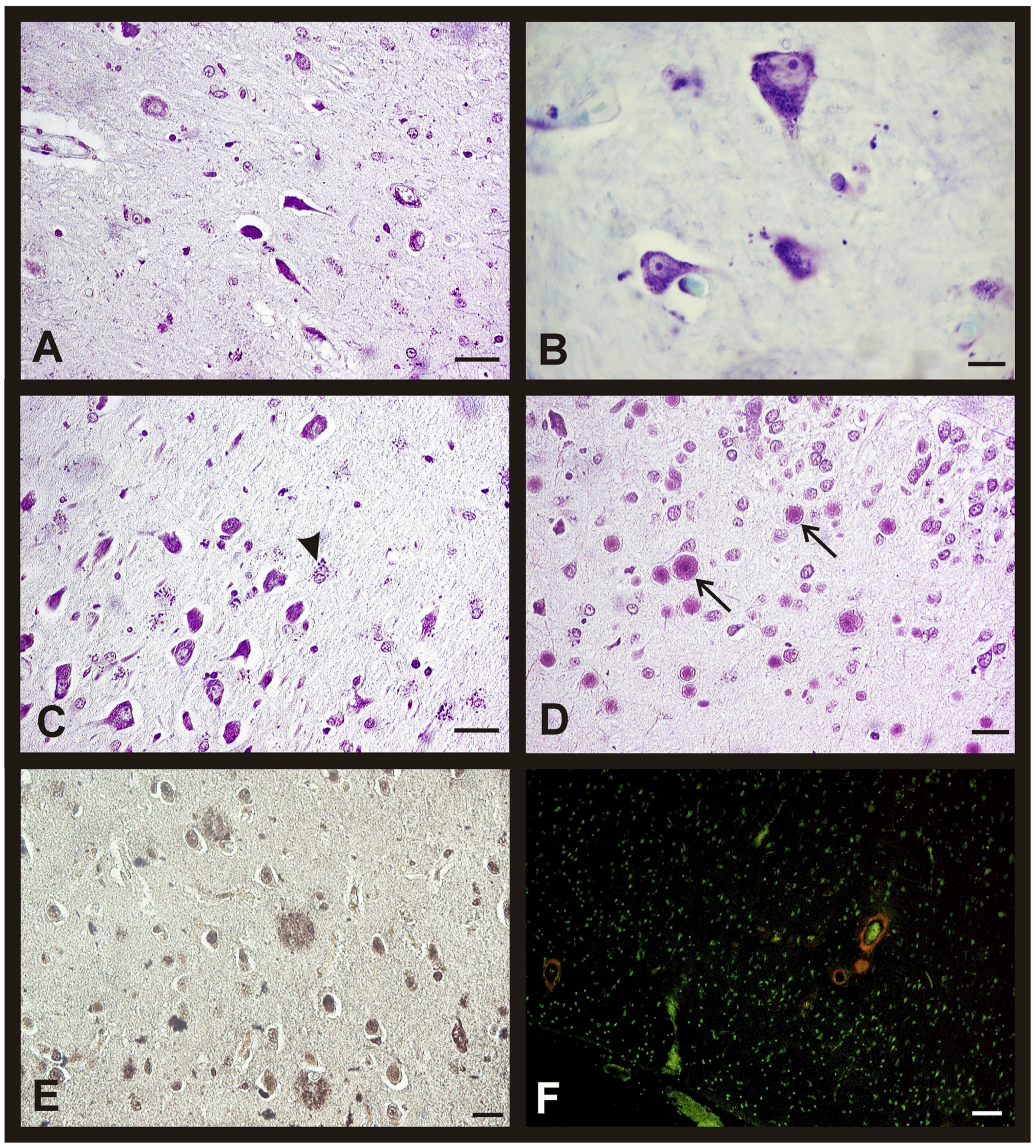
**Morphological changes in hippocampus and entorhinal cortex during aging in both sexes**. Representative microphotographs of human brain sections of non-pathological individuals contrasted with a Nissl method modification **(A–D)**, silver technique of Reusche **(E)** and a modification of Congo Red method **(F)**. **(A)** Shrinking neurons in the hippocampus of an 80 years old man. **(B)** Frontal cortex of a 75 years old woman, lipofuscin in neurons can be observed. **(C)** Hippocampus (CA3) of an 80 years old man, several astrocytes with lipofuscin granules are showed (arrowhead). **(D)** Hippocampus (CA4) of a 75 years old woman, several corpora amylacea can be observed (arrows). **(E)** Diffuse senile plaques in the entorhinal cortex of an 85 years old man. **(F)** Vessels with amyloid (red fluorescence) in frontal cortex of an 80 years old woman. Bars: **(A,C)**, 60 μm; **(B,D)**, 10 μm; **(E)**, 40 μm; **(F)**, 50 μm.

When we analyzed the morphological brain changes during aging in both sexes, we first observed some shrinking neurons in the different areas of the hippocampus in aged individuals. These cells undergo a wide range of modifications in their somas and nuclei that made them look larger and thinner, even in some cases, due to the intensity of the nuclear staining, it was impossible to distinguish the nucleolus (Figure 3A). Moreover, we often observed lipofuscin granules in non-degenerating neurons (Figure 3B).

The number of astrocytes increased with age in the entorhinal cortex, CA2, CA3, and CA4 while it was always scarce in the CA1 and *subiculum*. These glial cells also accumulated lipofuscin granules with age and their nuclei turned rounder and bigger than in the youngest subjects (Figure 3C). We also observed, mainly in the oldest individuals, corpora amylacea in contact to the pial surface, and in the CA4 close to the dentate gyrus (Figure 3D). The entorhinal cortex of some individuals of the over 80 group showed diffuse senile plaques (SP) and few isolated mature SP (Figure 3E), whereas the presence of NFT was not a common feature. In the subpial area, we found some vessels with a great extracellular deposit of amyloid protein (Figure 3F). It is important to note that these changes are not related with the gender of the subjects.

In the frontal cortex, we found the same morphological changes described previously for the hippocampus and the entorhinal cortex. In fact, we also found an increased neuronal content of lipofuscin and a higher number of corpora amylacea in contact with the pial surface in aged individuals. SP (mainly diffuse SP) were only observed in the oldest subjects who also showed inconstant perivascular amyloid deposits (data not shown). Once again, all these changes are related with age and not with gender.

### Neuronal density in Alzheimer's disease

**Figure 4:**
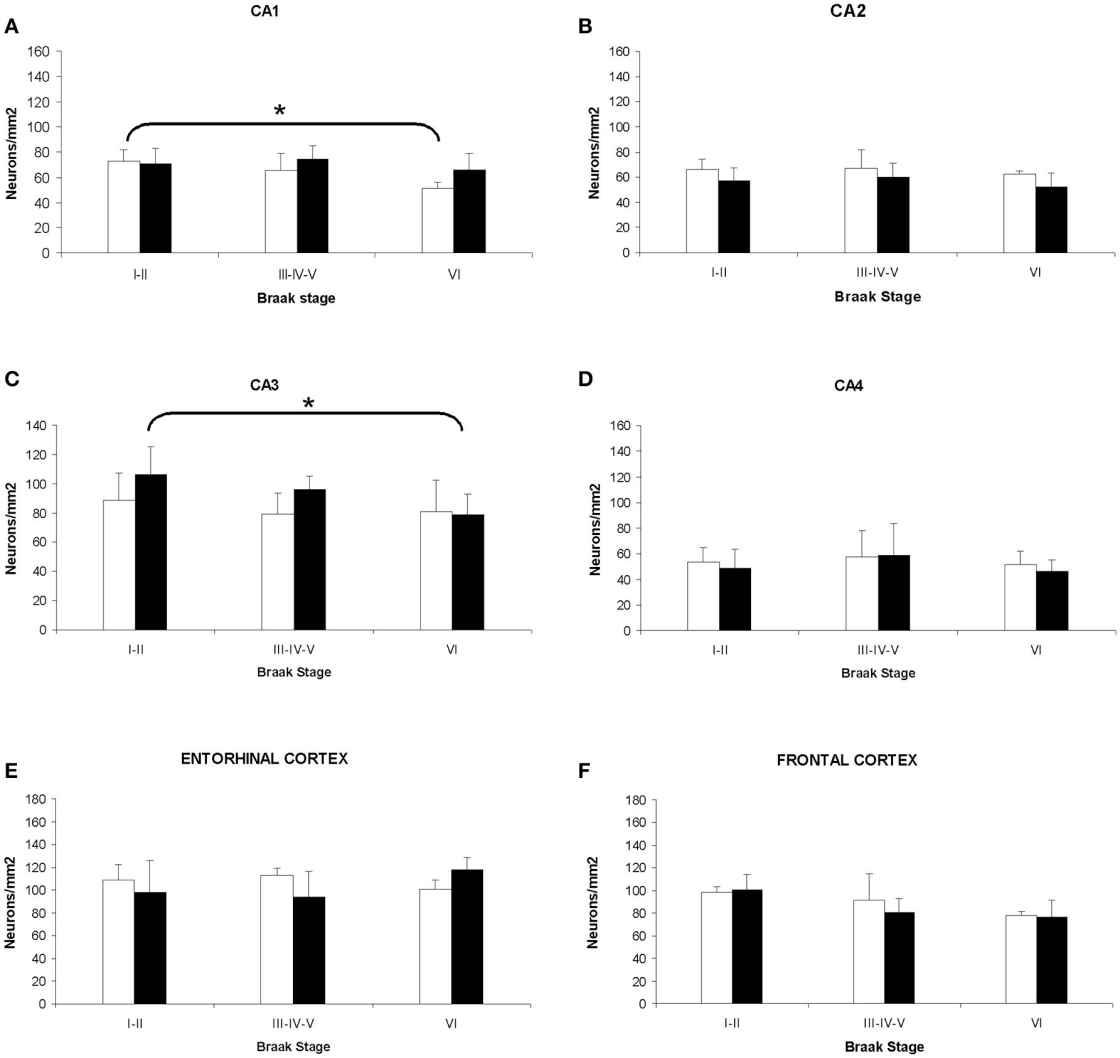
**Quantitative changes in neuronal density in certain hippocampal fields (CA1–4) and in frontal and entorhinal cortices between men and women during AD progression (I–VI Braak's stages)**. **(A)** CA1 hippocampal area. **(B)** CA2 hippocampal area. **(C)** CA3 hippocampal area. **(D)** CA4 hippocampal area. **(E)** Entorhinal cortex. **(F)** Frontal cortex. Bars represent mean density in a 20x field ± *SD* (*n* = 6). ^*^Statistically significant differences, *p* ≤ 0.05. Women, white bars; Men, black bars.

**Figure 5:**
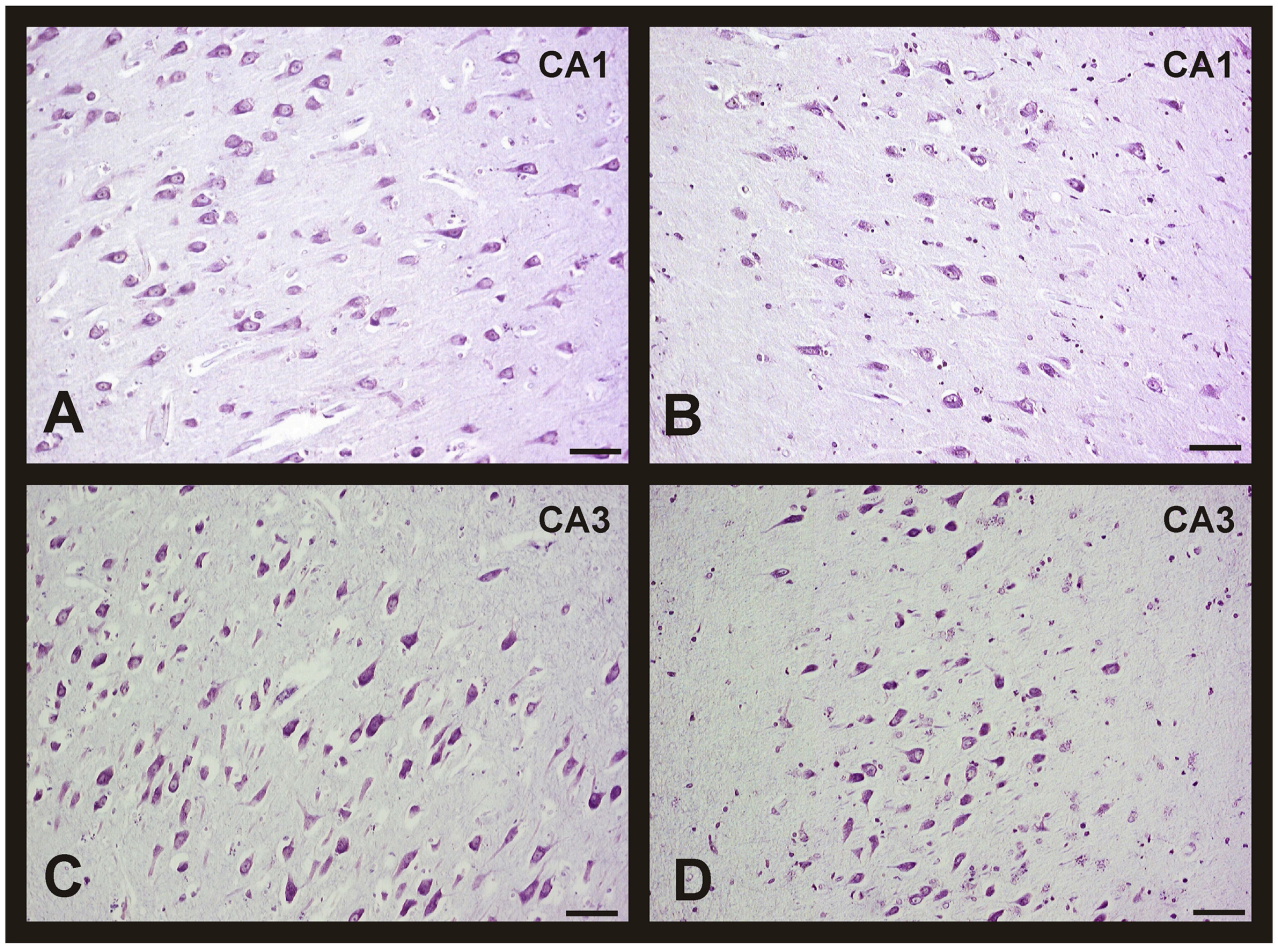
**Changes in neuronal density in hippocampus between men and women during AD progression (I-VI Braak's stages)**. Representative microphotographs of human brain sections of individuals with AD pathology, contrasted with a Nissl method modification. **(A)** Hippocampus (CA1) of a 70 years old woman (Braak's stage I). **(B)** Hippocampus (CA1) of a 75 years old woman (Braak's stage V). **(C)** Hippocampus (CA3) of a 75 years old man (Braak's stage I). **(D)** Hippocampus (CA3) of an 80 years old man (Braak's stage VI). Bar: 20 μm.

The analysis of cellular counting demonstrated that neuronal density in AD is not related with the gender or the degree of disease progression in the CA2, CA4, and entorhinal cortex, since significant differences were not found in these areas between sexes and Braak's stages (Figures 4B,D,E). In the CA1 hippocampal area, women displayed a decrease in the number of neurons per mm^2^ when we compared the initial with the advance stages of AD (Figures 4A, 5A,B), with a negative correlation between neuronal density and Braak's stage (*r* = −0.662, *p* < 0.01; Table 1). Meanwhile, men did not show significant differences in this area. In contrast, in the CA3 hippocampal area men but not women showed a statistical significant decrease in the neuronal density in the later stages of AD (Figures 4C, 5C,D), and a negative correlation between these two parameters (*r* = −0.662, *p* < 0.01; Table 1).

**Table 1:** **Pearson coefficient of correlation between neuronal density and Braak's staging**.

	**CA1**	**CA2**	**CA3**	**CA4**	**EC**	**FC**
**WOMEN**						
Braak's stage	^**^r = −0.662	0	0	0	0	^**^r = −0.537
**MEN**						
Braak's stage	0	0	^**^r = −0.662	0	0	^**^r = −0.514

CA1–4, Hippocampal areas; EC, entorhinal cortex; FC, frontal cortex;

** , correlation exists with p < 0.001; 0, no correlation; r, correlation coefficient.

In the frontal cortex, we found that both men and women showed a statistical significant decrease in the neuronal density in the later stages of AD (Figure 4F). Moreover, a clear negative correlation between the number of neurons per area analyzed and the progression of the pathology in both, women (*r* = −0.537, *p* < 0.01; Table 1) and men (*r* = −0.514, *p* < 0.01; Table 1), was detected.

### Morphological changes of hippocampus and entorhinal cortex in Alzheimer's disease

**Figure 6:**
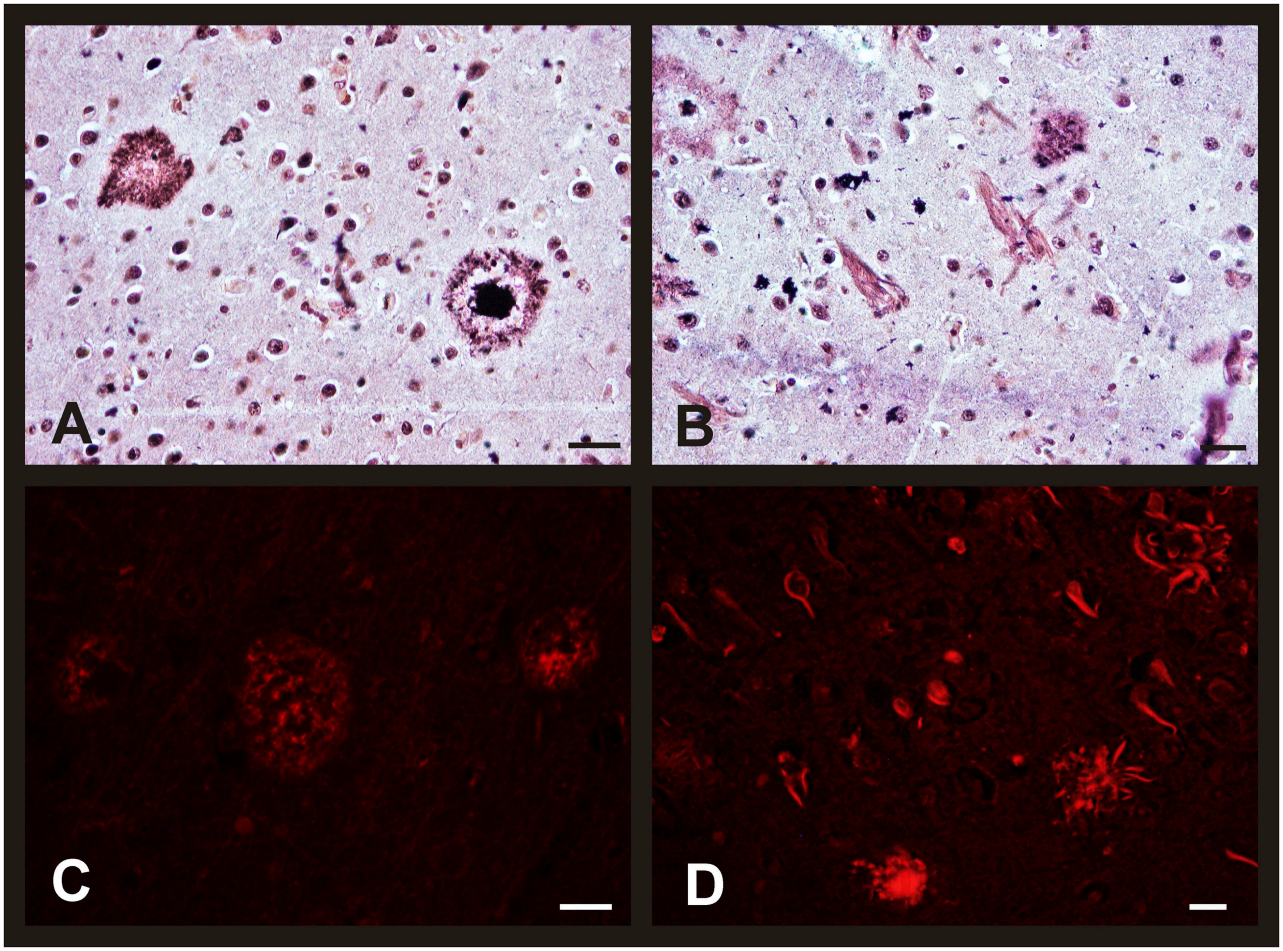
**Morphological brain changes in men and women during AD progression**. Representative microphotographs of human brain sections of individuals with AD pathology, contrasted with a silver technique of Reusche **(A,B)** and a modification of Congo Red method **(C,D)**. **(A)** Diffuse and mature plaques in the entorhinal cortex of an 80 years old man (Braak's stage II). **(B)** Senile plaques and neurofibrillary tangles in the hippocampus (CA2) of an 85 years old woman (Braak's stage VI). **(C)** Diffuse and mature plaques (red fluorescence) in the entorhinal cortex of an 80 years old man (Braak's stage II). **(D)** Senile plaques, in different maturation stages, and neurofibrillary tangles (red fluorescence) in the entorhinal cortex of an 80 years old man (Braak's stage VI). Bars: **(A,C)**, 60 μm; **(B)**, 10 μm; **(D)**, 60 μm.

The morphological brain changes observed in individuals with AD were similar to those previously described for the non-pathological cases over 80's (i.e., changes in neuronal features). As expected, the number of NFT and SP (as well as their level of development) was higher in AD subjects, increasing with the Braak's stage. Thus, we found diffuse isolated SP and some NFT in the entorhinal cortex of patients diagnosed with Braak's stage I-II. In advanced stages, the number of diffuse and mature SP with amyloid core increased notably in this brain area (Figures 6A,C). Finally, these characteristic AD hallmarks appeared in CA1 and CA2 (Figure 6B) and in a great number in the entorhinal cortex (Figure 6D) in the last stages of the disease.

Respect to the glia, we observed the presence of microglial cells, close to neuronal debris, in advanced stages of AD. Likewise, reactive astrocytes with pale big round nuclei and an important accumulation of lipofuscin granules were detected in all areas under analysis (except CA1 and *subiculum*) and in all Braak's stages. The number of corpora amylacea was higher in AD cases than in non-pathological ones.

In the frontal cortex of AD brains, we observed the same morphological changes as we did in the oldest subjects without pathology, but once again, lipofuscin accumulation as well as the presence of corpora amylacea and microglia seems to be higher in the AD samples. In the same way, the amount of amyloid protein and the number of SP and NFT are considerably higher in AD individuals than in those older subjects without pathology (data not shown). All the changes described here are common and similar to both sexes.

## Discussion

In this work, we look for changes in neuronal density during aging and in AD, in men and women, in some particularly vulnerable brain areas as hippocampus and entorhinal and frontal cortices.

Classical reports analyzing changes in neuronal density in two-dimensional space concluded that the percentage of substantial loss, which occurs with age, varies between 10 and 60 %, depending on the study and the neuronal population examined. Some studies, employing stereological-based sampling to derive estimates of cell number, reported that neuronal loss during aging is either undetectable or relative mild (West and Gundersen, 1990; Hof and Morrison, 2004). Our results are in concordance with these studies since we did not find a significantly neuronal loss with age. Importantly, we demonstrated a negative correlation between Braak's stage and neuronal density in some areas of AD brains. In this sense, West et al. concluded that neurodegenerative processes associated with normal aging and with AD are qualitatively different (West et al., 1994). A possible explanation could be that individuals without neurodegenerative pathology are able to mitigate the constant increased production of free radicals that naturally occurs with age, avoiding the neuronal loss. Interestingly, in several areas as frontal cortex middle-aged individuals showed less neurons per mm^2^ than older subjects did. We have to take into account that during human life exists a critical period around 55–70 years old where there is an important incidence of stroke, carcinogenic processes, and neurodegenerative diseases. In this sense, individuals who reach 80 years old are the ones that were able to throw this “bottleneck” and therefore could be the best adapted, known as “SuperAgers” (Harrison et al., 2012; Gefen et al., 2015). In this respect, some authors showed that these nonagenarians have immunological and antioxidant defenses and even episodic memory function equal or better than middle-aged individuals (Moroni et al., 2005; Harrison et al., 2012; Gefen et al., 2015).

Unlike aging, the loss of neurons in AD pathology may be due to a number of features that could facilitate or prone neurodegeneration. In this sense, the presence of SP, resulted from the abnormal extracellular accumulation and deposition of the amyloid-β peptide (40 or 42 amino acids), seems to promote neuronal death (Serrano-Pozo et al., 2011a). Several studies have demonstrated that mature SP are associated with deleterious effects on the surrounding neuropil i.e., increases of dystrophic neurites, recruitment and activation of astrocytes and microglial cells, synaptic loss, and neuronal death (Itagaki et al., 1989; Masliah et al., 1990, 1994; Pike et al., 1995; Knowles et al., 1999; Urbanc et al., 2002; Vehmas et al., 2003). Moreover, it is shown that the number of neurons per area is lower near to the mature SP than in distal areas in human and primate brains. The findings described by Shah et al. (2010) unequivocally reflect that while diffuse SP are commonly found in the brain of cognitive intact elderly people, dense-core plaques are most often in patients with AD dementia (Shah et al., 2010). In this work, we have observed that SP content increases as the disease progresses, which would explain the reduced neuronal density of these individuals.

Besides the SP, the presence of NFT, described as intraneural filamentous inclusions whose major constituent is aberrantly misfolded and abnormally hyperphosphorylated protein tau, correlates with the severity of the pathology and consequently with the neuronal loss in AD (Bierer et al., 1995; Gómez-Isla et al., 1997; Giannakopoulos et al., 2003), as we described in the Results section. However, whether NFT formation is a necessary precursor of the neuronal death or represents a protective response of damaged neurons in AD is still controversial.

Reactive astrocytes and activated microglial cells are commonly associated to dense-core SP, indicating, according with some authors, that amyloid-β peptide accumulation is the major trigger of this glial response (Itagaki et al., 1989; Pike et al., 1995; Vehmas et al., 2003). Our findings demonstrated an increase in this reactive cells with aging and with the Braak's stages. However, it has been recently described that the linear increase in reactive astrocytes and activated microglial cells through the entire disease course does not correlate with the amyloid deposition in the temporal associative isocortex (Serrano-Pozo et al., 2011b). Indeed, it has found a highly significant positive correlation between astrocytosis or microgliosis and NFT burden but not with amyloid concentration, suggesting that glial responses are related to neurofibrillary degeneration (Serrano-Pozo et al., 2011b).

As we have shown, there are little differences in neuronal density along aging in contrast to a clear loss of neurons in the progression of AD. According to our data, these differences not only depend on brain area but also on gender. In fact, in the CA3 field we found a decrease in the number of neurons per area in the non-pathological middle-aged men with respect to women. One possible explanation for this difference is the estrogen-mediated neuroprotection. Several authors pointed out that the high plasma levels of estradiol that pre-menopausal women show could exert a protective effect, preventing neuronal degeneration (Ishunina et al., 2007; Henderson, 2014). In this sense, in the first years after cessation of gonadal function women may show less accumulated damage. After menopause, women would become as vulnerable to neuronal degeneration as men, coinciding with the reduction in the estrogen production. In addition, sex differences were also found in the CA3 and CA4 hippocampal areas of the younger group. In these individuals, the neuronal density was higher in men than in women. This is in agreement with previous studies that show a higher neuronal content but a lower synaptic density in men than women in some brain areas (Allen and Gorski, 1991; Rabinowicz et al., 1999; Zaidi, 2010). It seems that the greatest amount of estrogens in brains of younger women could facilitate synaptic activity and optimize neuronal function, thus less neurons would be necessary to perform the same task (Aloisi et al., 1997; Brandt et al., 2013; Fester and Rune, 2015).

In AD subjects, a decrease in the density of neurons in relation to the stage of disease has been observed in the CA1 field in women but not in men. On the contrary, in the CA3 field, are men who lost more neurons. These observations are consistent with a region-specific neuronal loss in AD, which depends on the sex of the individuals and the stage of the pathology (Zaidi, 2010; Padurariu et al., 2012). The decrease in the neuronal population in AD may reflect the fact that the activation of antioxidant or compensatory mechanisms in both, women and men, cannot prevent neuronal loss in the brain. This phenomenon, together with the proven existence of sexual dimorphism in certain brain regions (Aloisi et al., 1997), could explain our results regarding gender differences in the pattern of neuronal degeneration in AD hippocampus; women seem to be more vulnerable in the CA1 area, while men in the CA3. So far, it has been described that the region-specific neuronal loss of this pathology occurs mainly in the CA1 brain area in both sexes (Adachi et al., 2003; Kerchner et al., 2010).

In summary, our findings clearly demonstrate that the neuronal loss that takes place during aging and to a greater degree in AD depends on the brain region and also varies between women and men. Therefore, our study highlights the need to consider the gender of the individuals in future studies to discriminate sexual variations in the pathological progression.

## Author contributions

EM-P contributed to writing the manuscript, performed and analyzed the neuronal density quantification. CO contributed to writing the manuscript and assisted in analysing the results and images processing. AN supervised the project and contributed to image processing. EdV performed part of staining procedures. JT contributed by formulating the initial hypothesis, directed the experiments performed and contributed to scientific discussions and manuscript writing.

## Funding

This work was supported by FISS, Instituto de Salud Carlos III and FEDER (Fondo Europeo de Desarrollo Regional) (PI15/00601) and Principado de Asturias (SV-PA-13-ECOEMP-80) grants.

### Conflict of interest statement

The authors declare that the research was conducted in the absence of any commercial or financial relationships that could be construed as a potential conflict of interest.

## Supplementary material

Supplementary Figure 1 **(A)** Schematic representation of hippocampal areas. **(B)** Representative microphotography of human hippocampus. DG, dentate gyrus.

## Abbreviations

AD: Alzheimer's disease
CNS: central nervous system
NFT: neurofibrillary tangles
SD: standard deviation
SP: senile plaques.
