# Supplementary figures and images for: Regional and Gender Study of Neuronal Density in Brain during Aging and in Alzheimer's Disease
Source: Front Aging Neurosci. 2016 Sep 13;8:213. doi: 10.3389/fnagi.2016.00213 (PMC5020132; doi:10.3389/fnagi.2016.00213)

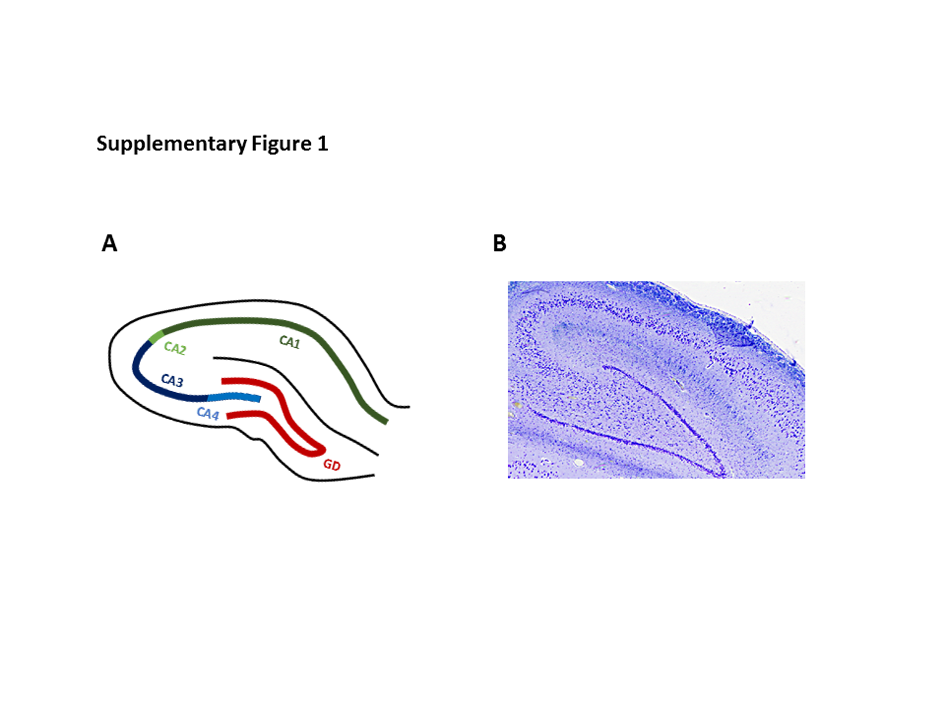

Supplement: Supplementary Figure 1 — (A) Schematic representation of hippocampal areas. (B) Representative microphotography of human hippocampus. DG, dentate gyrus. [file Image1.TIF]
